# Association Between Waist Circumference and the Prevalence of (Pre) Hypertension Among 27,894 US Adults

**DOI:** 10.3389/fcvm.2021.717257

**Published:** 2021-10-12

**Authors:** Jin-Yu Sun, Yang Hua, Hua-Yi-Yang Zou, Qiang Qu, Yue Yuan, Guo-Zhen Sun, Wei Sun, Xiang-Qing Kong

**Affiliations:** Department of Cardiology, The First Affiliated Hospital of Nanjing Medical University, Nanjing, China

**Keywords:** waist circumference, (pre) hypertension, abdominal obesity, fat distribution, body mass index

## Abstract

**Aims:** This study aimed to investigate the association between waist circumference and the prevalence of (pre) hypertension.

**Methods:** Cross-sectional data from the 2007–2018 National Health and Nutrition Examination Survey were analyzed. The historical trend of abdominal obesity was assessed by the Cochran–Armitage trend test. After preprocessed by the multiple imputation strategy, we used generalized additive models to assess the association of waist circumference with systolic/diastolic blood pressure and performed correlation analysis by the Spearman correlation coefficient. Moreover, we used multivariable logistic regression (non-adjusted, minimally adjusted, and fully adjusted models), restricted cubic spline, and sensitivity analysis to investigate the association between waist circumference and (pre) hypertension.

**Results:** A total of 27,894 participants were included in this study. In the fully adjusted model, waist circumference was positively associated with (pre) hypertension with odds ratios (95% confidence intervals) of 1.28 (1.18–1.40) in the young group and 1.23 (1.15–1.33) in the old group. Restricted cubic spline showed a higher prevalence of (pre) hypertension with the increase of waist circumference. In the subgroup analysis, waist circumference showed a robust trend across all BMI categories with odds ratios (95% confidence intervals) of 3.33 (1.29–8.85), 1.35 (1.17–1.57), 1.27 (1.13–1.41), and 1.09 (1.01–1.17) in underweight, normal weight, overweight, and obese individuals, respectively.

**Conclusion:** This study highlighted waist circumference as a significant biomarker to evaluate the risk of (pre) hypertension. Our results supported the measure of waist circumference regardless of BMI when evaluating the cardiometabolic risk related to fat distribution.

## Introduction

Obesity, together with the associated cardiovascular disorders, has posed a growing health threat with an estimated prevalence of 1.4 billion worldwide (1, 2). Excess weight gain is long recognized as a significant cause of hypertension, contributing to about 78% of the risk for primary hypertension in men and 65% in women (3). However, sizeable obese individuals remain free of hypertension during their lifetimes. Considering that abdominal fat accumulation is a higher-risk obesity phenotype than excess subcutaneous adipose tissue, studies suggested that body fat distribution might account for the heterogeneity of obesity (4–7).

Among the many techniques to evaluate body composition, body mass index (BMI) remains a primary method to estimate body fat and assess obesity-related cardiometabolic risk (8, 9). However, BMI fails to consider the body fat distribution and could not distinguish fat from lean mass. Therefore, BMI alone is inadequate to capture a full view of obesity-related cardiometabolic risk. Compared with BMI, waist circumference is strongly related to the absolute amount of abdominal fat (10, 11). Previous studies have demonstrated waist circumference as a risk factor for all-cause and cardiovascular-specific mortality beyond BMI (12, 13). A recent consensus statement by the IAS and ICCR Working Group highlighted the importance of waist circumference in clinical practice, considering the additional value in identifying the higher-risk obesity phenotype (11).

However, little is known about the relationship between waist circumference and (pre) hypertension risk. This study aimed to investigate the association between waist circumference and (pre) hypertension prevalence based on a representative United States population.

## Materials and Methods

### Data Source and Study Population

National Health and Nutrition Examination Survey (NHANES) is a publicly available database recording the health and nutritional status among the US population (https://www.cdc.gov/nchs/nhanes/index.htm). The NHANES database has been extensively analyzed in a series of publications in scientific and technical journals worldwide (14–16). All the involved participants were selected using a stratified multistage probabilistic sampling method to ensure a representative sample. This study used data from five cycles of the continuous NHANES (2007–2008, 2009–2010, 2011–2012, 2013–2014, 2015–2016, and 2017–2018). We included individuals with information on body measures, blood pressure, medical conditions, diabetes, smoking status, alcohol consumption, dietary interview, administration of anti-hypertensive medications, and standard biochemistry profile. The exclusion criteria were as follows: (1) pregnant individuals, (2) age < 18 or ≥ 80 years, (3) estimated glomerular filtration rate (eGFR) < 60 ml/min/1.73 m^2^, and (4) participants without BMI or waist circumference records. The National Center for Health Statistics Research Ethics Review Board has approved this study, and informed consent was obtained from all participants.

### Body Mass Index and Waist Circumference Measurement

Weight, height, and waist circumference were measured using standardized techniques and equipment by a trained examiner. BMI was calculated by dividing weight in kilograms by the square of their height in meters (kg/m^2^). Then, BMI was also converted into a categorical variable using WHO categories: underweight (<18.5 kg/m^2^), normal weight (18.5–24.9 kg/m^2^), overweight (25–29.9 kg/m^2^), class I obesity (30–34.9 kg/m^2^), class II obesity (35–39.9 kg/m^2^), and class III obesity (≥40 kg/m^2^) (17, 18). Waist circumference was measured directly against the skin at the superior lateral border of the iliac crests. The examiner stood on the right side of a participant, palpated the hip area to locate the right ilium of the pelvis, and marked a horizontal line above the most superior lateral border. Then, the examiner extended a steel tape around the waist at the measurement mark level, and the waist circumference data were taken after the subject exhaled one normal breath. Also, waist circumference was categorized into quartiles, and the lowest quartile (Q1) was set as the reference group.

### The Definition of (Pre) Hypertension

After resting in a seated position for 5 min, certified examiners measured the systolic blood pressure and diastolic blood pressure using a mercury sphygmomanometer following the recommended procedures by the American Heart Association. At least three consecutive blood pressure readings were obtained and reported as average systolic/diastolic blood pressure. In this study, we defined (pre) hypertension as (1) average systolic blood pressure ≥ 130 mmHg, (2) average diastolic blood pressure ≥ 85 mmHg, (3) current administration of prescribed anti-hypertensive medications, or (4) self-reported hypertension (19–21). The criteria of 130/85 mmHg was based on the International Society of Hypertension guideline, which is internationally acceptable from an epidemiological view (22).

### Covariates

Covariates were selected based on previously published studies on hypertension (19, 23, 24). We obtained age (continues), sex (male and female), race/ethnicity (non-Hispanic White, non-Hispanic Black, Mexican American, other Hispanic, and other races), and education levels (below high school, high school, and above high school) from the demographic questionnaire. Self-reported diabetes history (diabetes, borderline, or non-diabetes), smoking status (smoked at least 100 cigarettes in life or not), alcohol consumption (having at least 12 alcoholic drinks per year or not), and administration of anti-hypertensive medications (yes/no) were obtained from the health questionnaire. Individuals smoking more than 100 cigarettes during their lifetime were considered smokers (25), and participants consuming at least 12 alcohol drinks per year were considered alcohol users (26). Sodium intake (mg) was obtained from dietary interviews, whereas creatinine (mg/dl) was collected from laboratory tests. We calculated the eGFR based on the Chronic Kidney Disease-Epidemiology Collaboration (27). The detailed study design and procedures were provided on the NHANES website (https://wwwn.cdc.gov/nchs/nhanes/analyticguidelines.aspx).

### Statistical Analysis

Continuous variables were presented as mean ± standard deviation (normal distribution), median with Q1–Q3 (skewed distribution), or percentages (categorical variables). Kolmogorov–Smirnov test was used to assess the normality. Baseline characteristics were compared between individuals with and without (pre) hypertension using one-way ANOVA test (normal distribution), Kruskal–Wallis test (skewed distribution), or chi-square test (categorical variables). Importantly, to minimize bias caused by missing data, we applied multivariate multiple imputation strategies based on five replication and the Markov chain Monte Carlo method to fill the missing covariates (28, 29).

Distributions of waist circumference were illustrated by genders using kernel density estimation with Gaussian kernels. Then, we used generalized additive models to assess the association of waist circumference with systolic/diastolic blood pressure. Their correlation was also analyzed by the Spearman correlation coefficient. In addition, we illustrated the prevalence of obesity (BMI > 30 kg/m^2^) and abdominal obesity (waist circumference ≥ 102 cm in males and ≥88 cm in females) from 2007–2018. The historical trend was assessed by the Cochran–Armitage trend test.

Multivariable logistic regression analysis was used to estimate the association of waist circumference and BMI with (pre) hypertension. The models were stratified by age groups (young age: <45, and old age: ≥45) (30) and adjusted for BMI (only when analyzing waist circumference), waist circumference (only when analyzing BMI), age, sex, race/ethnicity, education levels, diabetes history, smoking status, alcohol consumption, height, and administration of anti-hypertensive medications. The odds ratios (ORs) with 95% confidence intervals (CIs) were calculated accordingly. To further illustrate the association between waist circumference and (pre) hypertension, we also conducted restricted cubic spline regression with five knots located at the 5th, 27.5th, 50th, 72.5th, and 95th percentiles and flexibly modeled the underlying relationship in males (reference set at 102 cm) and females (reference set at 88 cm), respectively (31). Sensitivity analyses were performed to examine whether the associations were modified by categories of BMI (underweight, normal weight, overweight, and obesity), eGFR (below median, and equal/above median), and sodium intake (below median, and equal/above median).

All statistical analyses were performed by the R software version 3.6.1 (The R Foundation for Statistical Computing). A value of *p* < 0.05 was considered statistically significant.

## Results

### Characteristics of the Study Population

We initially evaluated 32,304 individuals from NHANES 2007–2018. After excluding participants who were pregnant (*n* = 372), with eGFR <60 ml/min/1.73 m^2^ (*n* = 3,099), and without BMI (*n* = 29) or waist circumference (*n* = 910) data, a total of 27,894 participants were ultimately included. In the overall population, the prevalence of (pre) hypertension was 44.1%, the median age was 46 (32–60) years, the median waist circumference was 97.7 (87.2–109.1), and the median BMI was 28.2 (24.4–32.8) kg/m^2^. Among the 12,306 individuals identified with (pre) hypertension, 8,923 (72.5%) participants were with a self-reported history of hypertension, 10,254 (83.4%) were taking anti-hypertensive drugs, and 7,496 (60.9%) were with an average blood pressure of ≥130/85 mmHg. The detailed baseline characteristics are shown in Table 1.

**Table 1:** Characteristics of the study population.

	**Overall**	**(Pre) hypertension**	**Non-hypertension**	***P*-value**
N	27,894	12,306	15,588	
Age (years)	46 (32, 60)	57 (45, 66)	37 (27,50)	<0.001
Sex (female, %)	14,069 (50.4%)	5,823 (47.3%)	8,246 (52.9%)	<0.001
Waist circumference (cm)	97.7 (87.2, 109.1)	103.9 (94.2, 114.9)	92.7 (83.0, 103.5)	<0.001
BMI (kg/m^2^)	28.2 (24.4, 32.8)	30.1 (26.4, 34.9)	26.7 (23.2, 30.9)	<0.001
Body mass index (categories)				<0.001
Normal weight	7,538 (27.0%)	2,027 (16.5%)	5,511 (35.4%)	
Underweight	443 (1.6%)	86 (0.7%)	357 (2.3%)	
Overweight	9,066 (32.5%)	3,937 (32.0%)	5,129 (32.9%)	
Class I obesity	5,938 (21.3%)	3,193 (25.9%)	2,745 (17.6%)	
Class II obesity	2,821 (10.1%)	1,665 (13.5%)	1,156 (7.4%)	
Class III obesity	2,088 (7.5%)	1,398 (11.4%)	690 (4.4%)	
SBP (mmHg)	118.7 (108.0, 129.3)	131.3 (120.0, 141.3)	112.0 (105.3, 119.3)	<0.001
DBP (mmHg)	70.7 (64.7, 77.3)	74.0 (67.3, 83.3)	69.3 (62.7, 74.0)	<0.001
Race/ethnicity (%)				<0.001
Non-Hispanic White	10,885 (39.0%)	4,786 (38.9%)	6,099 (39.1%)	
Non-Hispanic Black	5,781 (20.7%)	3,176 (25.8%)	2,605 (16.7%)	
Mexican American	4,549 (16.3%)	1,758 (14.3%)	2,791 (17.9%)	
Other Hispanic	3,105 (11.1%)	1,290 (10.5%)	1,815 (11.6%)	
Other races	3,574 (12.8%)	1,296 (10.5%)	2,278 (14.6%)	
Education levels (%)				<0.001
Below high school	6,950 (24.9%)	3,285 (26.7%)	3,665 (23.5%)	
High school	6,473 (23.2%)	2,979 (24.2%)	3,494 (22.4%)	
Above high school	14,471 (51.9%)	6,042 (49.1%)	8,429 (54.1%)	
Diabetes (%)				<0.001
No/borderline	24,679 (88.5%)	9,837 (79.9%)	14,842 (95.2%)	
Yes	3,215 (11.5%)	2,469 (20.1%)	746 (4.8%)	
Smoking (yes, %)	12,077 (43.3%)	5,996 (48.7%)	6,081 (39.0%)	<0.001
Drinking (yes, %)	19,855 (71.2%)	8,614 (70.0%)	11,241 (72.1%)	<0.001
eGFR (ml/min/1.73m^2^)	132.8 (106.5, 165.5)	125.3 (97.9, 161.1)	138.0 (113.4, 168.5)	<0.001

*BMI, body mass index; SBP, systolic blood pressure; DBP, diastolic blood pressure; eGFR, estimated glomerular filtration rate*.

**Figure 1:**
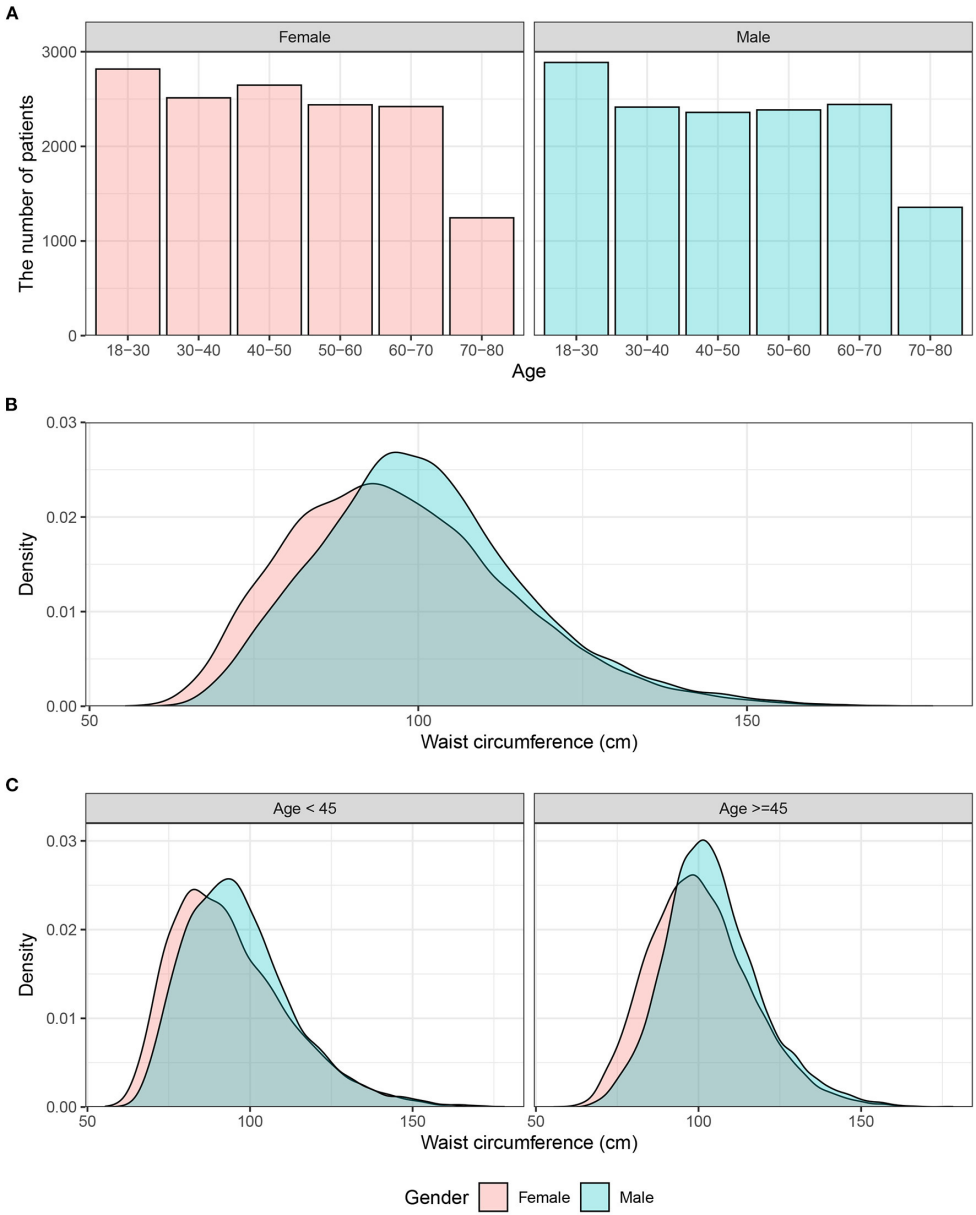
Distribution of waist circumference in males and females. **(A)** Histograms of age distribution in male and female individuals. **(B)** The overall distribution of waist circumference. **(C)** The distribution of waist circumference in individuals <45 years or ≥45 years old.

We illustrated the number of individuals across age groups (18–40, 40–60, 60–80) in Figure 1A, and the distribution of waist circumference stratified by sex is provided in Figures 1B,C. As shown in Supplementary Figures S1, S2, both BMI and waist circumference were positively and significantly correlated with systolic/diastolic blood pressure. Additionally, we illustrated the prevalence of obesity and abdominal obesity in Supplementary Figure S3. A significant increasing trend was observed in both obesity and abdominal obesity from 2007 to 2018.

### Association Between Body Mass Index and (Pre) Hypertension

In the minimally-adjusted model, increased BMI alone was significantly associated with (pre) hypertension in young (OR, 1.51; 95% CI, 1.46–1.56) and old individuals (OR, 1.44; 95% CI, 1.39–1.49). However, when introducing waist circumference as a continuous variable, we observed a weaker association between BMI and (pre) hypertension (Table 2). In the fully adjusted model, the ORs for BMI were 1.15 (1.04–1.27) and 1.15 (1.05–1.25) in the young and old groups, respectively.

**Table 2:** The association between body mass index and (pre) hypertension.

	**Non-adjusted model**		**Minimally adjusted model**		**Fully adjusted model**	
	**Odds ratio**	***P*-value**	**Odds ratio**	***P*-value**	**Odds ratio**	***P*-value**
**Age** **<** **45 years**						
BMI (Per 5 kg/m^2^)	1.54 (1.50–1.59)	<0.001	1.51 (1.46–1.56)	<0.001	1.15 (1.04–1.27)	0.006
BMI (Categories)						
Underweight	0.57 (0.35–0.90)	0.022	0.79 (0.473–1.25)	0.342	1.02 (0.61–1.62)	0.939
Normal weight	Reference		Reference		Reference	
Overweight	2.12 (1.87–2.40)	<0.001	1.78 (1.56–2.02)	<0.001	1.33 (1.14–1.56)	<0.001
Class I obesity	3.58 (3.14–4.08)	<0.001	3.00 (2.62–3.45)	<0.001	1.73 (1.40–2.12)	<0.001
Class II obesity	4.54 (3.90–5.29)	<0.001	3.90 (3.31–4.59)	<0.001	1.73 (1.30–2.29)	<0.001
Class III obesity	7.98 (6.82–9.35)	<0.001	7.20 (6.06–8.56)	<0.001	2.19 (1.45–3.19)	<0.001
**Age** ≥ **45 years**						
BMI (Per 5 kg/m^2^)	1.48 (1.43–1.52)	<0.001	1.44 (1.39–1.49)	<0.001	1.15 (1.05–1.25)	0.002
BMI (categories)						
Underweight	0.89 (0.64–1.24)	0.489	0.95 (0.66–1.34)	0.754	1.30 (0.90–1.86)	0.16
Normal weight	Reference		Reference		Reference	
Overweight	1.60 (1.46–1.75)	<0.001	1.52 (1.38–1.67)	<0.001	1.16 (1.03–1.30)	0.016
Class I obesity	2.40 (2.17–2.65)	<0.001	2.21 (1.98–2.47)	<0.001	1.31 (1.10–1.55)	0.002
Class II obesity	3.34 (2.97–3.89)	<0.001	3.08 (2.66–3.56)	<0.001	1.44 (1.14–1.83)	0.003
Class III obesity	5.02 (4.24–5.97)	<0.001	4.72 (3.94–5.68)	<0.001	1.58 (1.139–2.2)	0.006

*Minimally adjusted model, we adjusted for age, sex, race/ethnicity, education, diabetes, smoking, drinking, height, and administration of anti-hypertensive medications*.

*Fully adjusted model, we adjusted for waist circumference, age, sex, race/ethnicity, education, diabetes, smoking, drinking, height, and administration of anti-hypertensive medications*.

### Association Between Waist Circumference and (Pre) Hypertension

**Figure 2:**
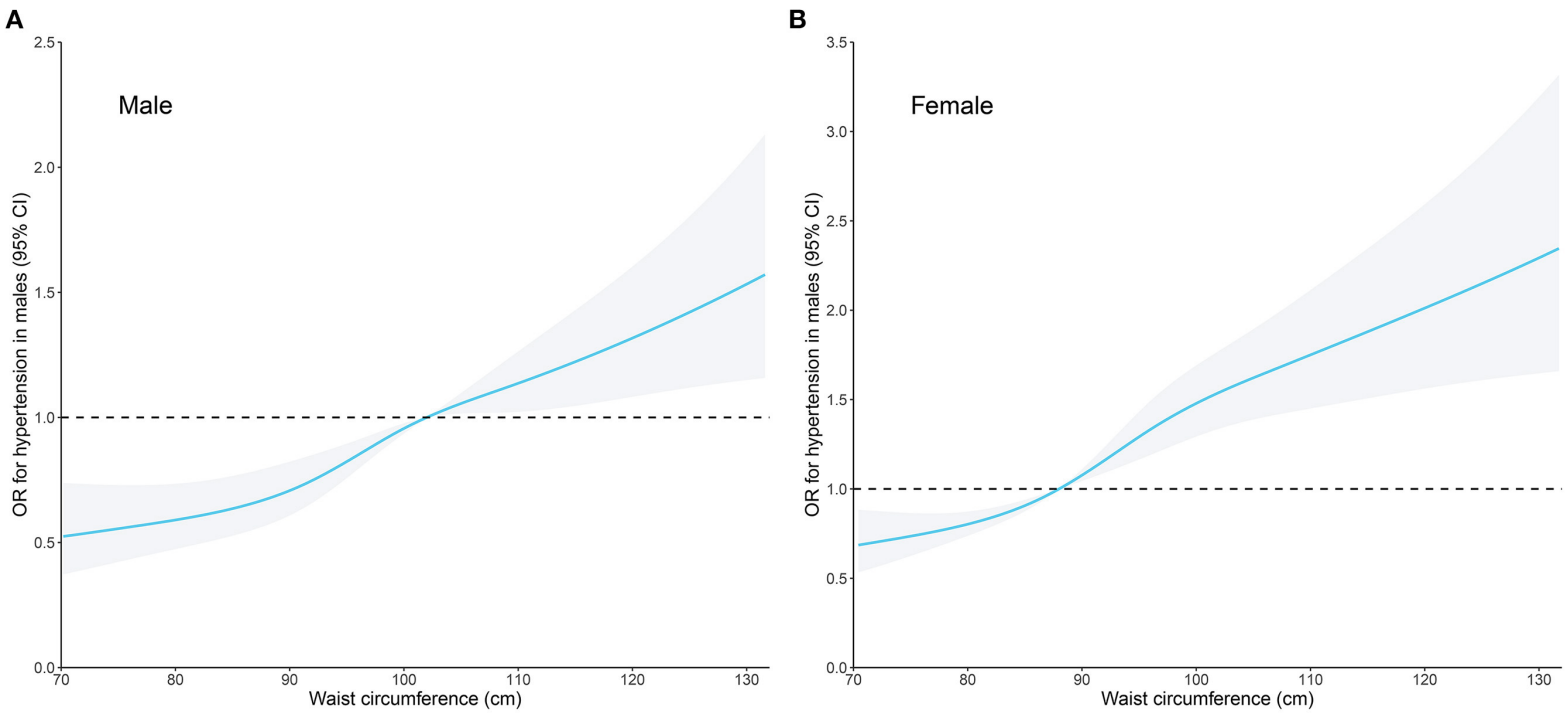
Restricted cubic spline plots of the association between waist circumference and (pre) hypertension in **(A)** males and **(B)** females. The association was adjusted for body mass index, age, sex, race/ethnicity, education levels, diabetes history, smoking status, alcohol consumption, height, and administration of anti-hypertensive medications. OR, odds ratio; CI, confidence intervals.

The increase in waist circumference showed a positive association with the prevalence of (pre) hypertension in all three models with ORs of 1.52 (1.48–1.56), 1.44 (1.40–1.48), and 1.28 (1.18–1.40) in young individuals, and ORs of 1.43 (1.34–1.47), 1.37 (1.34–1.41), and 1.23 (1.15–1.33) in old individuals, respectively. When fully adjusting for BMI, age, sex, race/ethnicity, education, diabetes, smoking, drinking, height, and administration of anti-hypertensive medications, individuals in the second to the fourth quartile displayed a significantly higher risk of (pre) hypertension in both the young and old groups (Table 3). In Figure 2, we used restricted cubic splines to visualize the relationship between waist circumference and (pre) hypertension across sexes. A consistent positive association was observed when adjusting for BMI, age, sex, race/ethnicity, education, diabetes, smoking, drinking, height, and administration of anti-hypertensive medications. Additionally, the non-adjusted restricted cubic splines are provided in Supplementary Figure S4.

**Table 3:** The association between waist circumference and (pre) hypertension.

	**Non-adjusted model**		**Minimally adjusted model**		**Fully adjusted model adjusted model**	
	**Odds ratio**	***P*-value**	**Odds ratio**	***P*-value**	**Odds ratio**	***P*-value**
**Age** **<** **45 years**						
Waist circumference (per 10 cm)	1.52 (1.48–1.56)	<0.001	1.44 (1.40–1.48)	<0.001	1.28 (1.18–1.40)	<0.001
**Categories**						
Q1	Reference		Reference		Reference	
Q2	2.22 (1.95–2.53)	<0.001	1.77 (1.55–2.03)	<0.001	1.35 (1.17–1.57)	<0.001
Q3	3.69 (3.25–4.21)	<0.001	2.73 (2.38–3.14)	<0.001	1.67 (1.41–1.99)	<0.001
Q4	6.98 (6.17–7.91)	<0.001	5.04 (4.41–5.77)	<0.001	1.99 (1.56–2.53)	<0.001
**Age** ≥ **45 years**						
Waist circumference (per 10 cm)	1.43 (1.34–1.47)	<0.001	1.37 (1.34–1.41)	<0.001	1.23 (1.15–1.33)	<0.001
**Categories**						
Q1	Reference		Reference		Reference	
Q2	1.59 (1.43–1.77)	<0.001	1.48 (1.32–1.65)	<0.001	1.18 (1.04–1.34)	0.008
Q3	2.47 (2.22–2.74)	<0.001	2.17 (1.93–2.43)	<0.001	1.41 (1.21–1.63)	<0.001
Q4	4.12 (3.69–4.59)	<0.001	3.38 (2.99–3.82)	<0.001	1.53 (1.24–1.88)	<0.001

*Q1, 55.5–87.2 cm; Q2, 87.2–97.7 cm; Q3, 97.7–109.1 cm; Q4, 109.1–178.2 cm*.

*Minimally adjusted model, we adjusted for age, sex, race/ethnicity, education, diabetes, smoking, drinking, height, and administration of anti-hypertensive medications*.

*Fully adjusted model, we adjusted for BMI, age, sex, race/ethnicity, education, diabetes, smoking, drinking, height, and administration of anti-hypertensive medications*.

### Sensitivity Analysis

**Figure 3:**
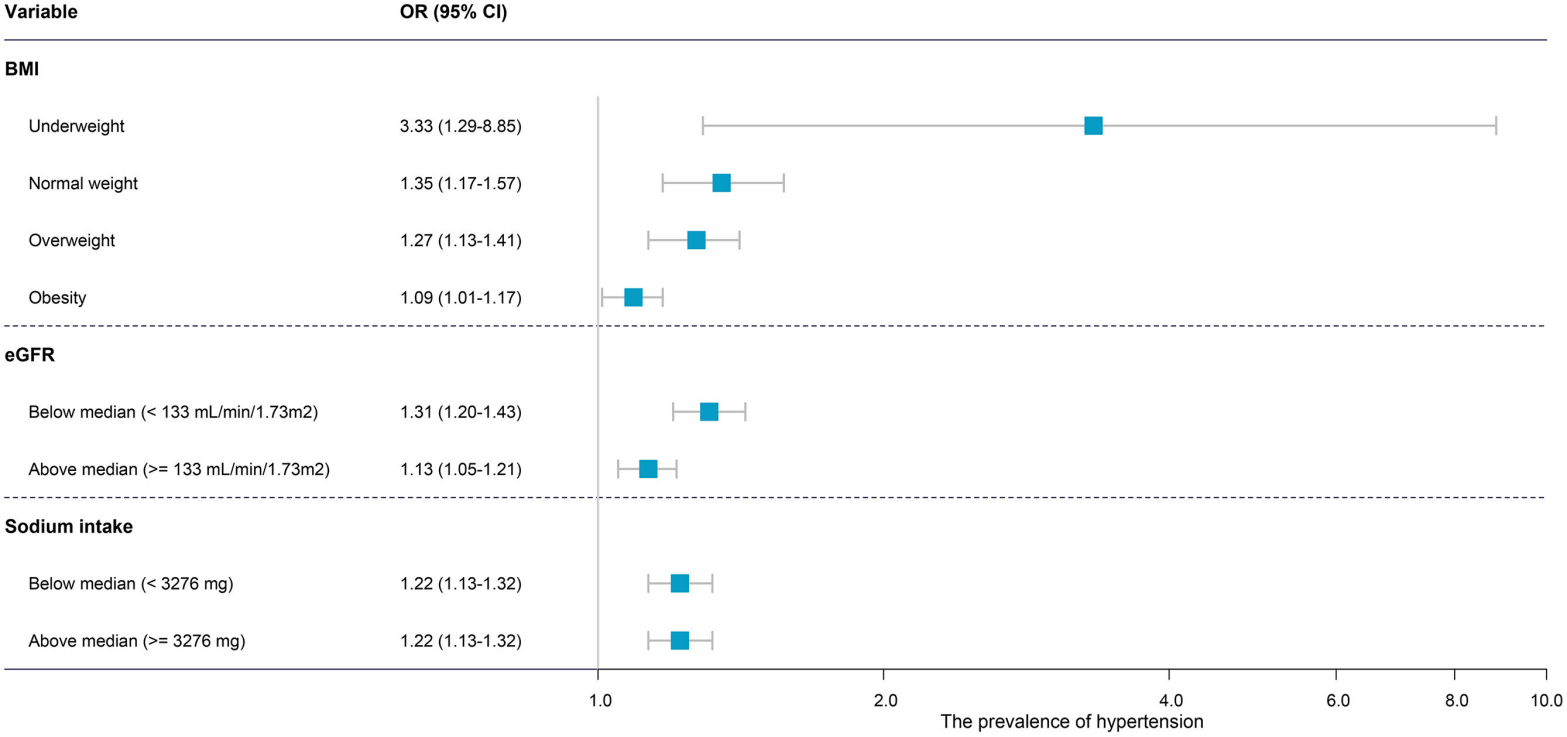
Forest plot of the subgroup analysis of the association between waist circumference and (pre) hypertension. The association was adjusted for body mass index, age, sex, race/ethnicity, education levels, diabetes history, smoking status, alcohol consumption, height, and administration of anti-hypertensive medications. OR, odds ratio; CI, confidence intervals.

For waist circumference, a robust trend was observed across all BMI categories with ORs of 3.33 (1.29–8.85), 1.35 (1.17–1.57), 1.27 (1.13–1.41), and 1.09 (1.01–1.17) in underweight, normal weight, overweight, and obese individuals, respectively (Figure 3). Also, when the cut-off value was set as 140/90 mmHg, the adjusted OR for waist circumference was 1.32 (1.17–1.49), which is similar to the cut-off value of 130/85 mmHg (OR, 1.22; 95% CI: 1.15–1.29). Moreover, we analyzed the influence of BMI in underweight, normal weight, overweight, and obesity individuals. The results showed ORs of 0.11 (0.01–0.80), 0.96 (0.74–1.25), 1.29 (1.04–1.60), and 1.24 (1.14–1.34), respectively (Supplementary Figure S5).

## Discussion

Emerging evidence indicates that abdominal adiposity has become a more severe problem beyond obesity defined by BMI alone worldwide (32). The waist circumference of Canadians experienced a 1.1-cm increase in males and 4.9 cm in females for an adjusted BMI from 1981 to 2007 (33), and the consistent alteration was observed in the United States, England, China, and Mexico (34). Currently, waist circumference is a preferred tool to evaluate abdominal fat accumulation compared with BMI or waist–hip circumference ratio, which shows a more robust association with the absolute amount of intra-abdominal or visceral fat (10, 35). Abdominal obesity, defined by waist circumference, significantly increased the adverse health risk with or without adjusting BMI (36).

However, a few studies investigated the effect of abdominal fat on hypertension, and the association between waist circumference and hypertension remains vague. Recently, a cross-sectional study analyzed the association of body mass index and waist circumference with hypertension based on 785 Algerian adults (37), but this study focused on blood pressure, and the researchers did not quantify the effect size on hypertension. The small sample size (*n* = 785) and limited race distribution (all Algerian adults) also reduced the reliability. Besides, all participants came for consultation due to an abnormal metabolic profile (instead of the normal population), which presumably resulted in additional selection bias. In another cross-sectional study in Turkey, Çam and Ustuner Top (38) analyzed the association of BMI and WC with hypertension based on 896 high school students. The prevalence of hypertension was significantly higher in obese and overweight adolescents than those with normal weight (1.6 and 15.5 vs. 6.2%, respectively). Logistic regression was performed to investigate the effect of BMI and WC on hypertension. It was surprising to find that BMI was independently associated with the prevalence of hypertension in both males and females, while the positive correlation was not observed in waist circumference. Moreover, Zhao et al. (39) conducted a cohort study on 10,265 non-hypertensive participants from rural China to clarify the relationship between gain in WC and the incidence of hypertension. After a 6-year follow-up, 2,027 hypertension cases occurred. After stratifying participants into four groups based on WC gain of baseline (≤-2.5%, −2.5-2.5%, 2.5-5%, and >5%), the relative risk for hypertension was significantly higher in those with WC gain >5% (for males, relative risk = 1.34, 95%CI = 1.15–1.57; for females, relative risk = 1.28, 95% CI = 1.10–1.50) compared with the reference group (−2.5-2.5%) after adjustment for BMI and lipid levels, indicating strong and positive role of waist circumference in hypertension.

Our results showed that the adverse effect of waist circumference remains after adjusting for BMI. Therefore, waist circumference is a robust obesity-associated cardiometabolic biomarker regardless of overall weight. When individuals share similar waist circumference, a higher BMI value suggests the relative increase in subcutaneous adipose tissue or muscle mass. Alternatively, for those with similar BMI but higher waist circumference, they usually represent a phenotype with increased deposition of intra-abdominal/visceral fat, decreased amount of subcutaneous adipose tissue/muscle mass, or both. Interestingly, a stronger association between waist circumference and (pre) hypertension was observed in individuals with a BMI of <25 kg/m^2^. A higher proportion of abdominal subcutaneous/intra-peritoneal fat in the total fat tissue could partly explain the difference (40). Although several guidelines recognize the importance of waist circumference in managing obesity, waist circumference is generally recommended to measure only in individuals with overweight or obesity. Waist circumference has not been included in global obesity surveillance so far, which might lead to a weakened recognition of obesity-associated cardiometabolic risk. Previous studies on the association between obesity and hypertension were based on BMI without considering visceral or retroperitoneal fat (41, 42). This study indicated that waist circumference should be measured regardless of BMI. A low waist circumference should be an important target for managing obesity-related metabolic disorders, even when the BMI has reduced to 25 kg/m^2^ or lower. Moreover, sarcopenic obesity (low BMI but high waist circumference) is a high-risk geriatric syndrome with a growing prevalence, which involves sex-related hormonal alteration, inflammatory response, and complex cellular mechanisms (43). Our results indicated the potential key role of sarcopenic obesity in the development of hypertension; therefore, the association between sarcopenic obesity and hypertension should be a focus of further investigation.

In our study, both waist circumference and BMI showed a stronger association with hypertension in young adults compared with the elderly. The age-related difference has been reported in previous studies (44–47). For example, in a cross-sectional study based on the Korean National Health and Nutrition Survey, waist circumference and BMI were reported to increase the risk of hypertension. The association was stronger in adults aged 19–39 years than those aged 40–64 years or >65 years across all BMI and waist circumference categories (44). However, the biological mechanisms underlying the age-related difference remain controversial. Hypertension is common in the elderly due to aging-induced vascular elasticity degeneration and peripheral vascular resistance (48). In contrast, hypertension in the young seems to be more attributed to a complex mixed impact of congenital genetic background, living conditions at birth or in early childhood, unhealthy living habits, or diverse comorbidities (49). Interestingly, some studies suggested that the difference might be partly caused by the low exposure of excess fat accumulation in young adults, which may predispose them to a vulnerable state to metabolic changes (45, 46, 50). Moreover, differences in fatty acids, secretion of angiotensinogen, and sympathetic nervous system activation between the young and the elderly may also play a role in age variation (51). The prevalence of hypertension in young adults has grown to be a severe health problem. It was estimated that one in seven youths in the United States had elevated blood pressure or hypertension (52). Accordingly, our study suggested that waist circumference should be measured and properly managed in young adults to prevent obesity-related adverse outcomes.

The current sex-specific thresholds of abdominal obesity were set as a waist circumference of ≥102 cm in males and ≥88 cm in females, widely used in many studies and guidelines (35). However, the cut-off points were initially converted from BMI based on a cross-sectional study in the white population. The waist circumference of 102 cm in males and 88 cm in females corresponded to the BMI threshold for obesity (30.0 kg/m^2^). Therefore, the current waist circumference threshold was initially designed to replace BMI as an alternative obesity biomarker without considering its unique advantage in estimating disease risk. Our results supported establishing a novel waist circumference classification system from a cardiometabolic view in concert with BMI, which could fully exert their unique superiority in managing obesity-related disease risk. Additionally, the relationship between obesity and resistant hypertension is an interesting and clinically significant topic. Hypertension resistance to more than two antihypertensive drugs is more frequently observed in the obese individuals compared with lean ones. In the following research, we will further investigate whether high circumference could be used to predict resistant hypertension and even as a therapeutic target.

Several advantages in this study should be highlighted. First, different from previous studies (53), we analyzed the continuous scales of waist circumference instead of the currently used cut-offs (≥102 cm in males and ≥88 cm in females), which provides a more comprehensive view of the association between waist circumference and (pre) hypertension. Second, the diagnostic criteria of 130/85 mmHg from the International Society of Hypertension guideline were adopted in our study. The use of recently proposed criteria gives additional value to our conclusion and allows us to evaluate the effect of waist circumference on (pre) hypertension, considering the disease burden. Third, to comprehensively investigate the role of waist circumference in evaluating hypertension risk, many covariates were involved in this study, especially BMI and height. Previous studies have suggested that the effect of abdominal obesity (by waist circumference) on adverse events could be fully understood only after adjusting for BMI (12, 54). Additionally, waist circumference varies among individuals with different heights, and short stature is correlated with an increased risk of cardiovascular diseases (55). Fourth, the large sample size (about 30,000 individuals) improved the statistical power and reliability of the result. Importantly, to avoid selection bias by excluding patients with missing data, we used multiple imputation to fill missing covariates following Dong and his colleagues (56).

Despite the novel insights into waist circumference and the prevalence of (pre) hypertension, some limitations should be noted. First, different ethnicities show varying diet, physical activity, genetic variation, lipid metabolism, and susceptibility to cardiovascular diseases. However, this study only used nationally representative US samples. The generalizability of our conclusion to other populations remains unclear. Second, our study revealed the association between waist circumference and the prevalence of (pre) hypertension. Nevertheless, it is difficult to determine the causality due to the inherent nature of a cross-sectional study. More prospective studies are required to ensure whether the increased waist circumference would result in an elevated risk of developing hypertension. Third, although we involved multiple covariates, we could not exclude the influence of other confounding factors completely. Fourth, waist-to-hip circumference ratio is another practical and easily measured biomarker for fat distribution or abdominal obesity, while only about 15% of individuals collected hip circumference. Due to a large amount of missing data, the analysis based on hip circumference would be unreliable. Fifth, this study aimed to investigate the association in the general population. Therefore, patients with comorbidities (e.g., heart failure, coronary heart disease, stroke, and diabetes) were also included. Still, including patients with comorbidities might result in potential bias. Additionally, sodium intake was estimated based on dietary assessment, which is relatively unreliable at the individual level than the gold standard. It should be further validated whether sodium intake modifies the association between waist circumference and (pre) hypertension using precise sodium intake evaluation methods.

## Conclusion

This study highlighted the waist circumference as a significant biomarker to evaluate the risk of (pre) hypertension. Our results supported the measure of waist circumference regardless of BMI when evaluating the cardiometabolic risk related to fat distribution.

## Data Availability Statement

The datasets presented in this study can be found in online repositories. The names of the repository/repositories and accession number(s) can be found in the article/Supplementary Material.

## Ethics Statement

The studies involving human participants were approved by National Center for Health Statistics Research Ethics Review Board. The patients/participants provided their written informed consent to participate in this study.

## Author Contributions

J-YS, G-ZS, WS, and X-QK conceived and designed the study. J-YS, YH, QQ, and YY analyzed the data. J-YS, H-Y-YZ, and YH wrote the paper. All authors provided critical revisions to the manuscript and approved the final manuscript.

## Funding

This study was supported in part by the Postgraduate Research and Practice Innovation Program of Jiangsu Province (SJCX21_0626), the National Key Research and Development Program of China (No.2019YFA0210100), and China International Medical Foundation (Z-2019-42-1908).

## Conflict of Interest

The authors declare that the research was conducted in the absence of any commercial or financial relationships that could be construed as a potential conflict of interest.

## Publisher's Note

All claims expressed in this article are solely those of the authors and do not necessarily represent those of their affiliated organizations, or those of the publisher, the editors and the reviewers. Any product that may be evaluated in this article, or claim that may be made by its manufacturer, is not guaranteed or endorsed by the publisher.

## Supplementary Material

Supplementary Figure S1 Smooth trajectories of the association of body mass index **(A,B)** and waist circumference **(C,D)** with systolic/diastolic blood pressure. Participants with waist circumference below 2.5th centile or above 97.5th centile were excluded.

Supplementary Figure S2 The heatmap of the correlation analysis using the Spearman correlation coefficient.

Supplementary Figure S3 The historic prevalence trend of obesity (BMI > 30 kg/m^2^) and abdominal obesity (waist circumference ≥ 102 cm in males and ≥88 cm in females) from 2007 to 2018.

Supplementary Figure S4 The non-adjusted restricted cubic spline plots of the association between body mass index and (pre) hypertension in **(A)** males and **(B)** females.

Supplementary Figure S5 Forest plot of subgroup analysis of the association between body mass index and (pre) hypertension. The association was adjusted for waist circumference, age, sex, race/ethnicity, education levels, diabetes history, smoking status, alcohol consumption, height, and administration of anti-hypertensive medications. OR, odds ratio; CI, confidence intervals.

## Abbreviations

BMI: body mass index
NHANES: National Health and Nutrition Examination Survey
eGFR: estimated glomerular filtration rate
OR: odds ratio
CI: confidence interval.
