# Supplementary figures and images for: Association Between Waist Circumference and the Prevalence of (Pre) Hypertension Among 27,894 US Adults
Source: Front Cardiovasc Med. 2021 Oct 12;8:717257. doi: 10.3389/fcvm.2021.717257 (PMC8545886; doi:10.3389/fcvm.2021.717257)

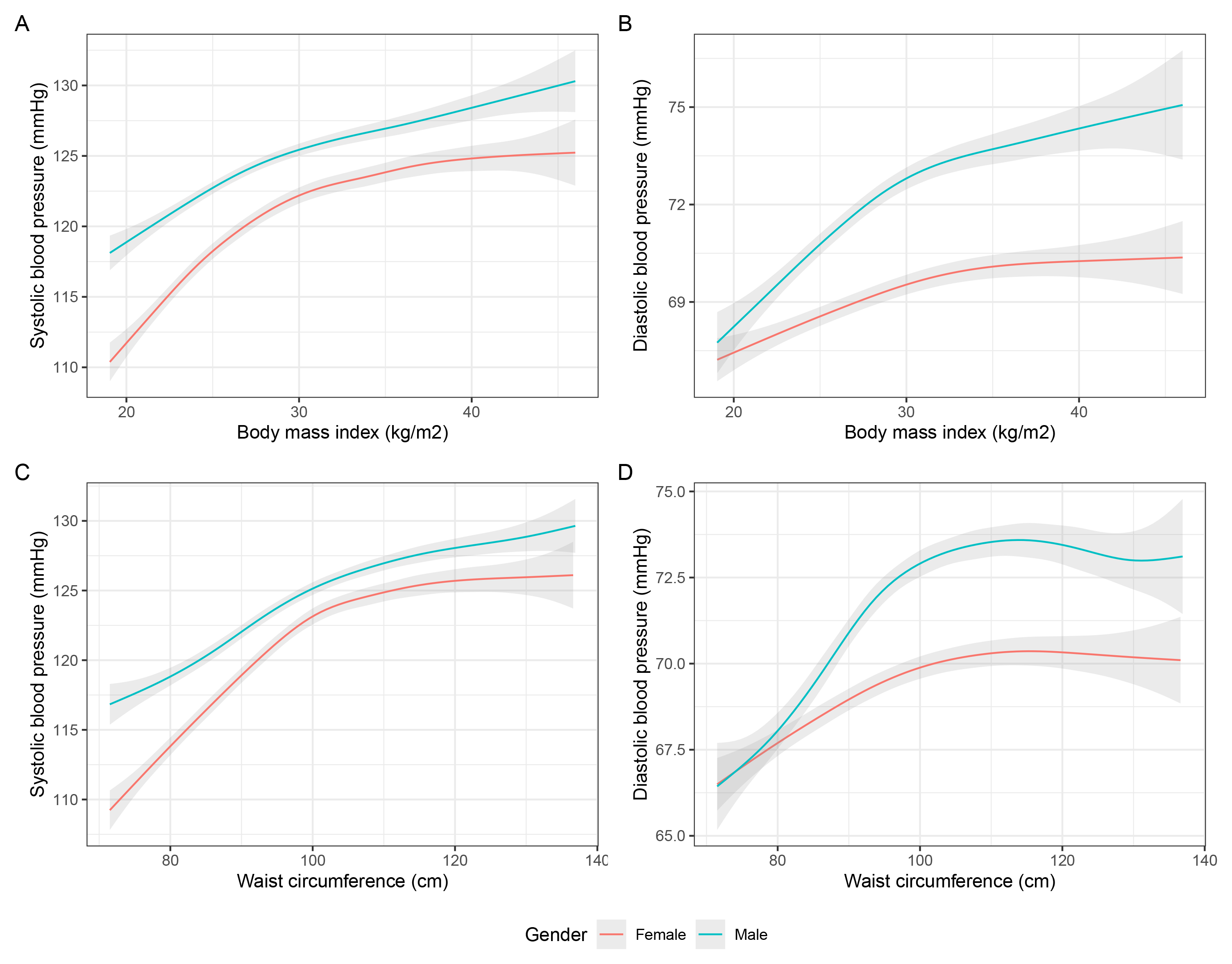

Supplement: Supplementary Figure S1 — Smooth trajectories of the association of body mass index (A,B) and waist circumference (C,D) with systolic/diastolic blood pressure. Participants with waist circumference below 2.5th centile or above 97.5th centile were excluded. [file Image_1.TIF]

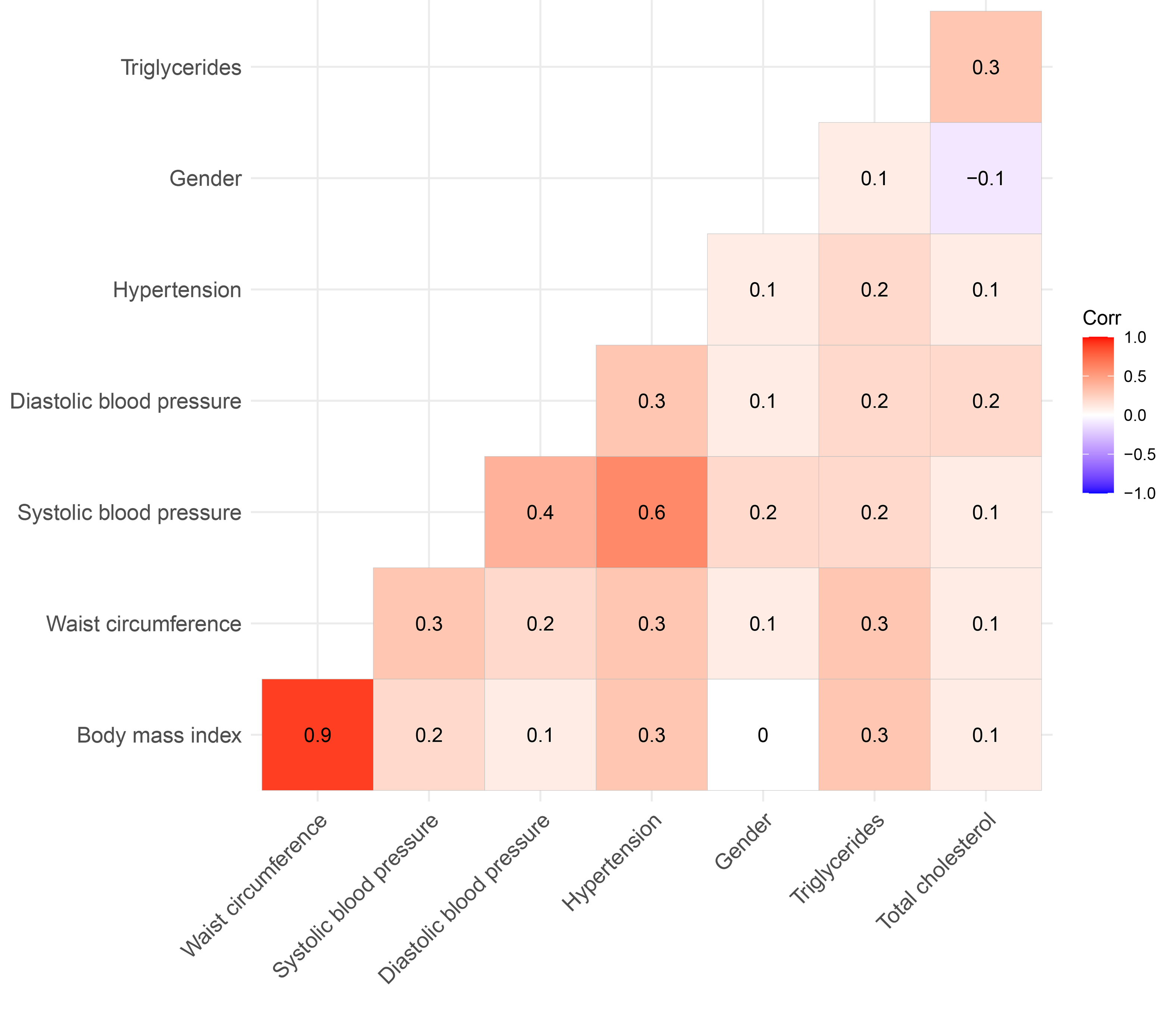

Supplement: Supplementary Figure S2 — The heatmap of the correlation analysis using the Spearman correlation coefficient. [file Image_2.TIF]

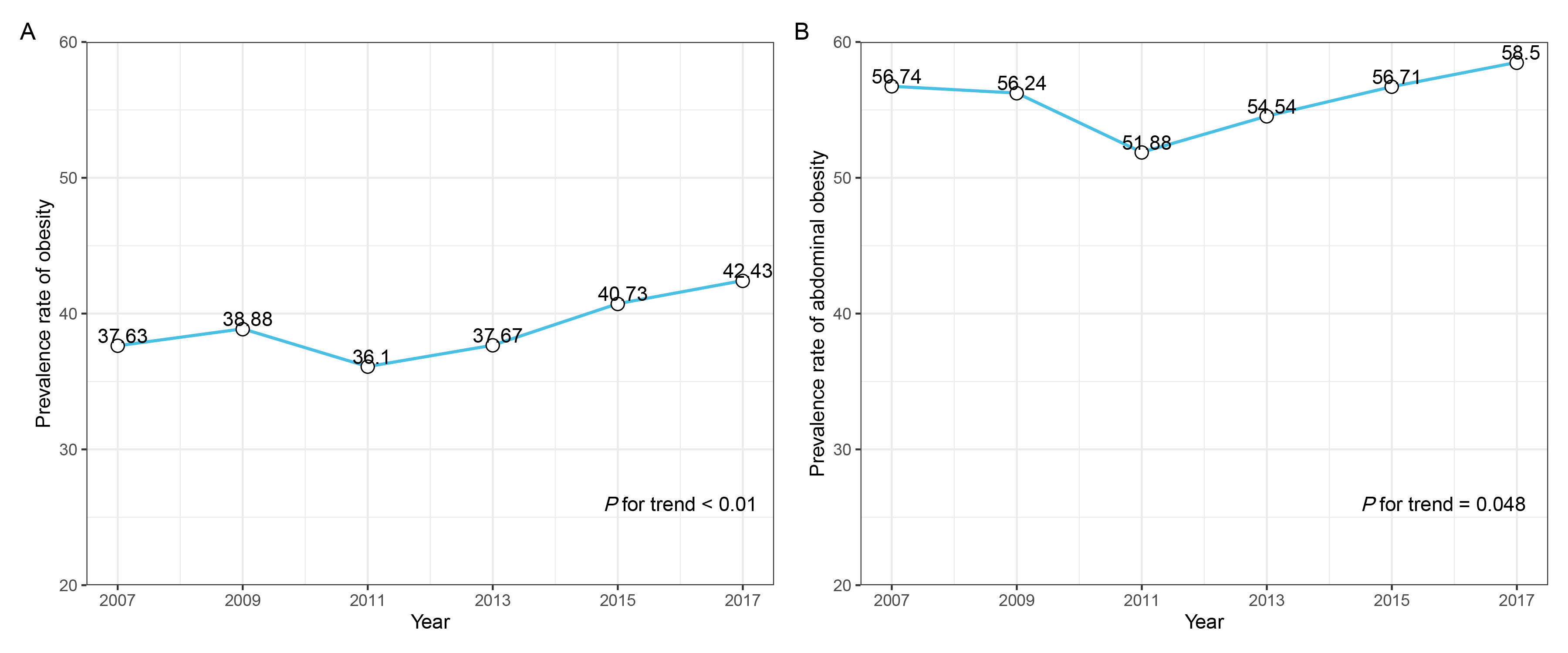

Supplement: Supplementary Figure S3 — The historic prevalence trend of obesity (BMI > 30 kg/m2) and abdominal obesity (waist circumference ≥ 102 cm in males and ≥88 cm in females) from 2007 to 2018. [file Image_3.TIF]

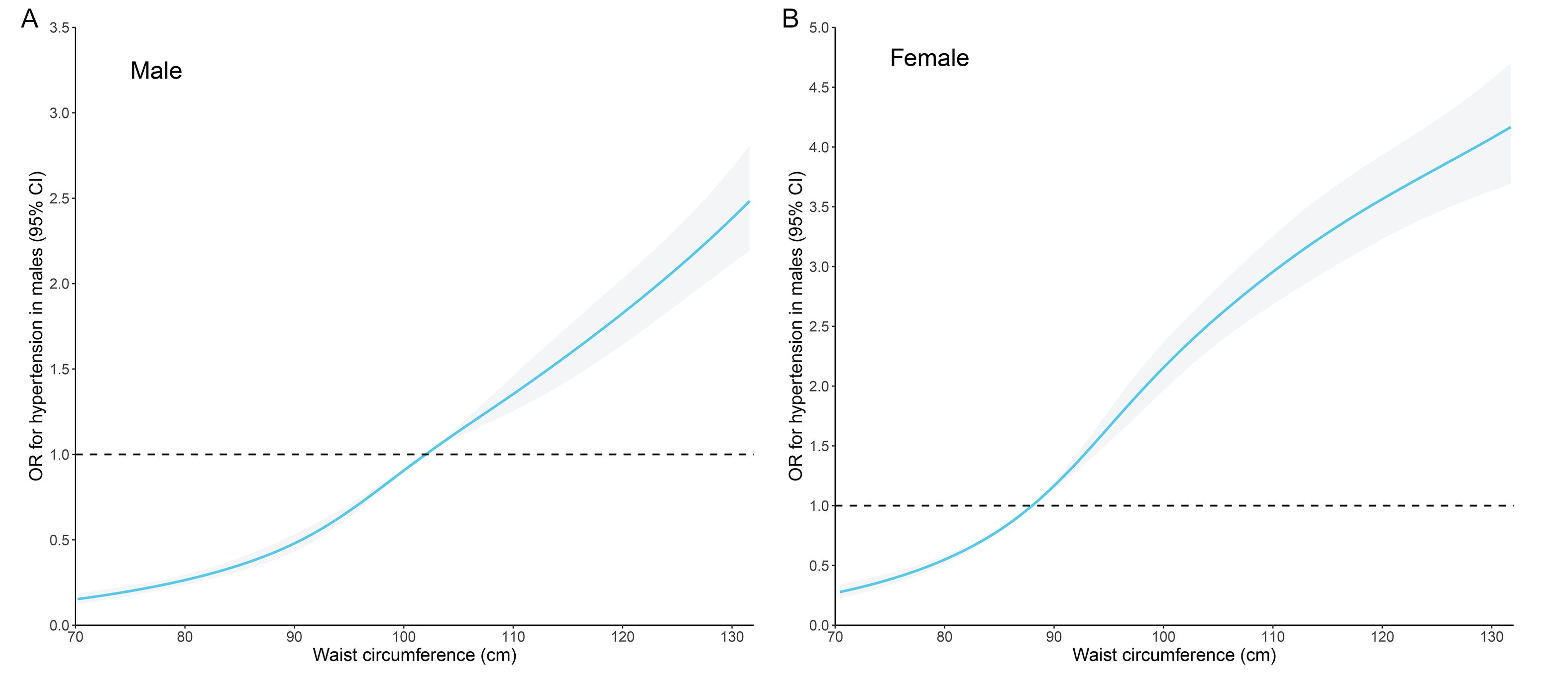

Supplement: Supplementary Figure S4 — The non-adjusted restricted cubic spline plots of the association between body mass index and (pre) hypertension in (A) males and (B) females. [file Image_4.TIF]

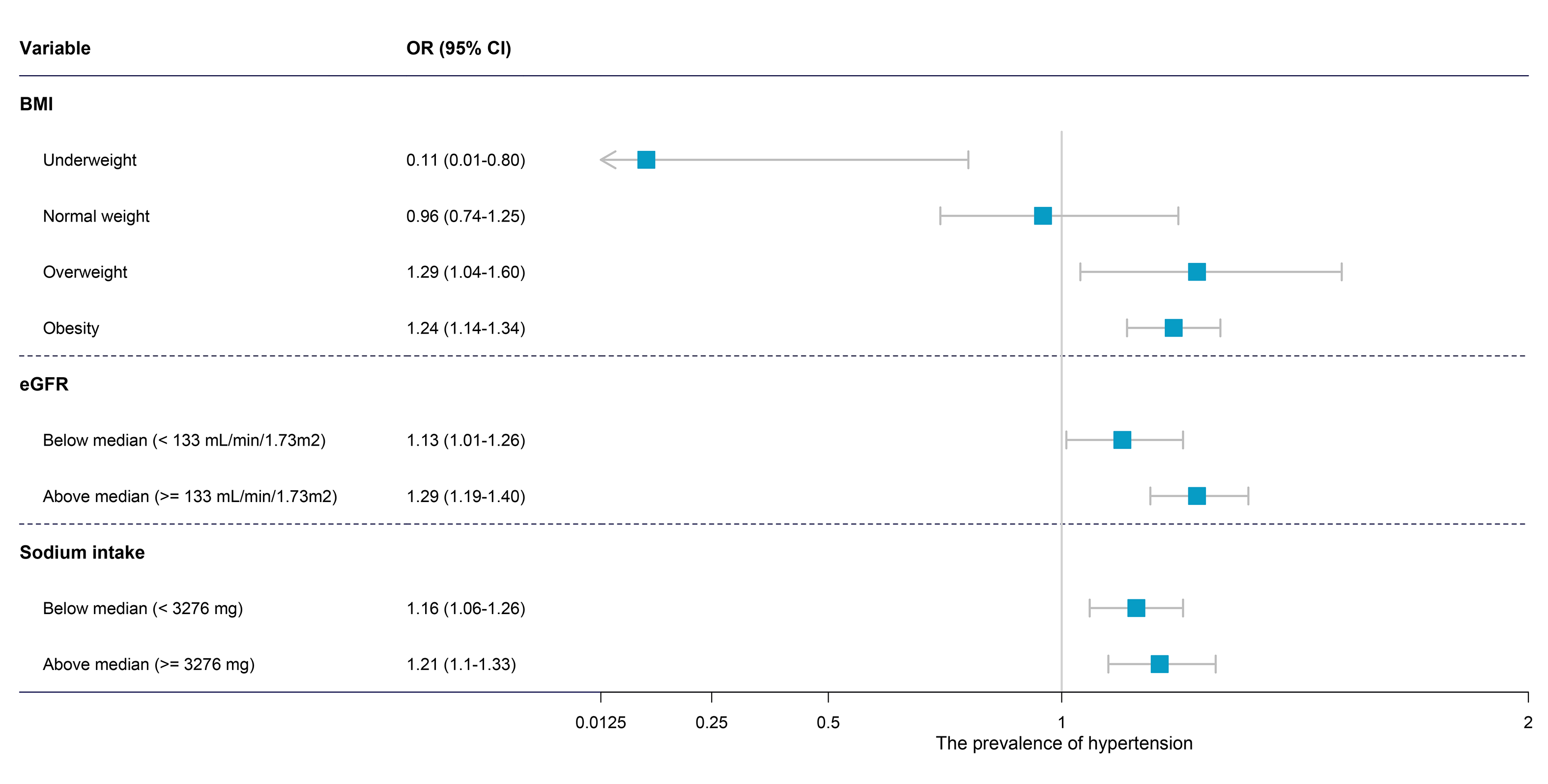

Supplement: Supplementary Figure S5 — Forest plot of subgroup analysis of the association between body mass index and (pre) hypertension. The association was adjusted for waist circumference, age, sex, race/ethnicity, education levels, diabetes history, smoking status, alcohol consumption, height, and administration of anti-hypertensive medications. OR, odds ratio; CI, confidence intervals. [file Image_5.TIF]
